# Long-chain polyunsaturated fatty acid (LC-PUFA) status in severe preeclampsia and preterm birth: a cross sectional study

**DOI:** 10.1038/s41598-021-93846-w

**Published:** 2021-07-19

**Authors:** Rima Irwinda, Rabbania Hiksas, Aprilia Asthasari Siregar, Yudianto Budi Saroyo, Noroyono Wibowo

**Affiliations:** 1 Maternal Fetal Division, Department of Obstetrics and Gynaecology, Faculty of Medicine, Universitas Indonesia/Cipto-Mangunkusumo Hospital, Jakarta, Indonesia; 2 Faculty of Medicine, Universitas Indonesia/Cipto-Mangunkusumo Hospital, Jakarta, Indonesia

**Keywords:** Lipids, Reproductive disorders, Outcomes research, Nutrition

## Abstract

Long-Chain Polyunsaturated Fatty Acid (LCPUFA) is essential throughout pregnancy, since deficiency of LPUFA may linked to obstetrical complications. This study aimed to investigate LCPUFA status in severe preeclampsia and preterm birth. A cross sectional study was conducted in 104 pregnant women, which divided into normal pregnancy, severe preeclampsia and preterm birth groups. Serum percentage and concentration of total LCPUFA, omega-3, alpha-linolenic acid (ALA), eicosapentaenoic acid (EPA), docosahexaenoic acid (DHA), omega-6, linoleic acid (LA), and arachidonic acid (AA) were measured using gas chromatography/mass spectrometry. Receiver operating characteristic (ROC), bivariate and multivariate analysis were performed. Severe preeclampsia showed the highest concentration of total PUFA and the lowest DHA percentage, with significantly higher Omega-6/Omega-3 ratio (*p* = 0.004) and lower omega-3 index (*p* < 0.002) compared to control. Preterm birth showed the least omega-3 concentrations, with significantly low omega-6 derivates (LA (*p* = 0.014) and AA (*p* = 0.025)) compared to control. LCPUFA parameters have shown to increase the risk in both conditions, particularly ALA ≤ 53 µmol/L in preeclampsia with OR 5.44, 95%CI 1.16–25.42 and preterm birth with OR 4.68, 95%CI 1.52–14.38. These findings suggest that severe preeclampsia and preterm birth have an imbalance in LCPUFA status.

## Introduction

Long-chain polyunsaturated fatty acid (LCPUFA) is essential in pregnancy. It is advised to take at least 200 mg/day of DHA in pregnancy which should be given earlier than 16 weeks^1^. Adequate intake on LCPUFA in periconception and during pregnancy is important to support adequate fetal growth and development, specifically brain development^2,3^. LCPUFA has a role in maternal health as dietary omega-3 has been correlated with reduced risk in poor maternal outcome associated with inflammation process^4^.

Obstetrical complication such as preeclampsia and preterm birth have been reported to associated with inflammation process. Pathogenesis of preeclampsia is hallmarked by abnormal placental development by placental ischemia which lead to inflammatory response and further widespread maternal endothelial dysfunction^5,6^. In preterm birth, oxidative stress and inflammation initiating an environment suitable for labor such as uterine contractility, cervical ripening, and membrane ruptures by releasing proinflammatory cytokines which normally initiated in term labor^7,8^.

Preeclampsia and preterm birth are still a major worldwide burden for maternal–fetal health. Hypertensive disorder may occur in about 10% of pregnancies^9^. Particularly in Indonesia, hypertension disorders of pregnancy impacted 3.3% of population and become a major cause of maternal death^10^. With regard of preterm birth, Indonesia was accounted for the top five countries with the highest rate of preterm birth worldwide in 2014^11^. In addition, nutrient deficiencies are also a major problem among pregnant women in Indonesia. Our study showed most of first trimester pregnant women has low level of iron, zinc, and vitamin D^12^. Also, a study about LCPUFA status in first trimester of pregnancy showed that most of pregnant women in Indonesia had low intake and blood concentrations of LCPUFA, including LA, AA, ALA, EPA, and DHA^13^.

With numbers of researchers showing the benefits of LCPUFA in maternal and fetal health, there is no study in LCPUFA status in obstetric complication in pregnant women in Indonesia, particularly in preeclampsia and preterm birth. Therefore, this study aimed to evaluate LCPUFA blood concentration status in preeclampsia and preterm population in Indonesia.

## Materials and methods

### Study participants

This was a cross sectional study of total 104 pregnant women. Normal pregnancy (n = 44) was compared to pregnancy with severe preeclampsia (n = 30) and preterm birth (n = 30). The analysis of variance sample size estimation was used among 3 groups to obtain type I error at least 5% and power as high as 80%^14^. From previous studies, we found that mean ± SD for EPA + DHA percentage in preterm birth was 1.41 ± 0.81^15^ and in preeclampsia was 2.69 ± 2.56^16^, which results in calculated effect size (Cohen’s D) 0.67 and minimum sample requirement was 25. Accounting for dropout, minimum 30 samples for each group were used in this study.

Data were obtained in Cipto Mangunkusumo National Referral Hospital, Budi Kemuliaan Hospital, and Koja Regional Hospital, in Jakarta from January to December 2019. This study has been approved by Ethical Committee for Research in Human from the Faculty of Medicine, Universitas Indonesia (KET-628/UN2.F1/ETIK/PPM.00.02/2019). All of the participants have given their informed consent prior to their inclusion to the study. The study confirmed to the principles set out in the World Medical Association Declaration of Helsinki.

Preeclampsia with severe feature was defined by any of the findings of severe features based on American College of Obstetricians and Gynecologists (ACOG) 2013 criteria; blood pressure ≥ 160/110 mmHg (on two occasions at least 4 h apart), thrombocytopenia (platelet count less than 100.000/microliter), progressive renal insufficiency (serum creatinine > 1.1 mg/dL or doubling serum creatinine in the absence of other renal disease), new onset cerebral or visual disturbances, and pulmonary edema^17^. Spontaneous preterm birth group were deliveries in ≥ 26 weeks to < 37 weeks with no maternal morbidities during pregnancy. The exclusion criteria in this study include multiple pregnancy, congenital anomalies, preterm premature rupture of membrane, diabetes mellitus, infection, autoimmune diseases.

Basic characteristics of subjects were descripted, including maternal age, maternal blood pressure, gestational age, mode of delivery, pre-pregnancy body mass index (BMI), and birth weight. Term gestational age defined for ≥ 37 weeks. Birth weight < 2500 classified as low and ≥ 2500 as normal to high. The calculated BMI was the pre-pregnancy BMI, and categorized based on Asia–Pacific BMI criteria which includes underweight (< 18.5), normal (18.5–22.9), overweight (23–24.9), and obese (≥ 25).

### Sample preparation and fatty acids quantification

Non-fasting maternal blood samples were obtained soon after delivery, and excluded if the delivery had been more than 1 h. Samples were collected using venous puncture into 5-mL tubes (Vacutainer; Becton–Dickinson). The serum was directly separated out from the whole blood, then frozen at − 80 °C until further analysis.

For fatty acids measurement, serum was firstly thawed at room temperature for about 30 min, then extracted using (choloform:methanol, 2:1). 150 uL of Methanol (Merck, Germany) was added to 100 uL serum for protein precipitation and homogenized thoroughly and then added with 300 uL Chloroform (Merck, Germany) contained Nonadecanoic Acid Methyl Ester (Supelco) as internal standard and vortexed. The mixture then centrifuged at 2500 g in 5 min and the chloroform layer which contain fatty acids was transferred to different tubes. The chloroform phase was then dried with gentle nitrogen stream and then reconstituted with n-hexane (Merck, Germany). Tetramethyl Ammonium Hydroxide (TMAH) in methanol (Sigma) was added and incubated 2 h in room temperature for trans-esterification while vortexed, upper phase then transferred to Gas Chromatography (GC) vials^18–22^.

Fatty Acid calibration curve prepared by diluting stock of FAME Mix, C4-C24, (Cat. 18919-1AMP, Lot. LC16765V, Supelco, Bellefonte, PA, USA) with n-Hexane (Merck, Germany) to obtained 50 mg/mL in concentration. Concentration of every fatty acids then converted to umol/L based on their molecular weight. Further dilution was done to obtain working stock and the serial diluted with h-Hexane to make 9 levels of calibration. FAME Mix standard contained Myristic (C14:0), Palmitic (C16:0), Palmitoleic (C16:1 w7), Stearic (C18:0), Oleic (C18:1 w9), Linoleic (C18:2 w6), y-linolenic (C18:3 w6), α-Linolenic (C18:3 w3), AA (C20:4 w6), DGLA (C20:3 w6), EPA (C20:5 w3), DHA (C22:6 w3). Mass spectrometer was set to Selected Ion Monitoring (SIM) Mode and the ions monitored for every fatty acids as follows: C14:0 for 199 m/z, C16:0 for 199 m/z, C16:1 for 194 m/z, C18:0 for 298 m/z, C18:1 for 296 m/z, C18:2 for 294 m/z, C18:3 w6 for 292 m/z, C18:3 w3 for 292 m/z, C20:4 w6 for 203 m/z, C20:3 w6 for 222 m/z, C20:5 w3 for 201 m/z, and C22:6 w3 for 199 m/z. The quality control of samples showed the intra- and interassay coefficients of variations (CV) range for all fatty acids were 1.0–16.1%. Based on CLSI, maximum CV within the calibration rate was 15%, and if it near the detection limit was 20%^18–22^.

Standards and samples were injected to 7890 Gas Chromatography (GC) System (Agilent Technologies, USA) coupled with 5977 Mass Selective Detector (MSD) (Agilent Technologies, USA) with Electron Impact (EI) source. Fatty Acids separated through HP-88 column (30 m × 0.25 mm × 0.25 um film thickness) (Agilent Technologies, USA). GC oven temperature was set to ramping from 50 to 180 °C with 7.07 °C rate per minute and 180 °C to 230 °C with 7.07 °C rate per minute. MSD was set in SIM mode. The quality control substance was a pooled serum 2 level based on triglyceride concentration. The accuracy of the measurement was performed as much as 3 levels, with 103–115% recovery rate^18–22^.

### Statistical analysis

The extracted data for the characteristics of subject including maternal age, blood pressure, gestational age, mode of delivery, body mass index, and birth weight. The serum LCPUFA was measured both percentage and concentration, including total LCPUFA, omega-3, alpha-linolenic acid (ALA), eicosapentaenoic acid (EPA), docosahexaenoic acid (DHA), omega-6, linoleic acid (LA) and arachidonic acid (AA).

Data were analysed using Statistical Package for Social Sciences (SPSS) version 25.0 (IBM, United States). The numeric data were firstly checked for normal distribution using Kolmogorov Smirnov test, then presented as mean ± SD if normally distributed and as median (IQR) if not normally distributed. One-way ANOVA and Kruskal Wallis test were used to identify significances among groups. Post hoc tests were performed to locate those specific differences in each group, using Bonferroni and Games-Howell test after One-way ANOVA and Mann Whitney test after Kruskal Wallis.

The Receiver Operating Characteristic (ROC) curve analyses were used to analyse corresponding cut-off values, sensitivity and specificity of each LCPUFA concentration variables. The overall performance of each parameter for predicting either preeclampsia or preterm birth were assessed by estimating the area under the curve (AUC). The cut off values for parameters with high overall performance were determined at the point on the curve with the highest value of sensitivity and specificity. Bivariate analysis was performed using Chi-Square test, followed by multivariate association between each LCPUFA serum variables and each pregnancy outcomes in separate logistic regression models after adjusting maternal age, gestational age (only available for preeclampsia), and pre-pregnancy BMI. All results corresponding to *p*-values < 0.05 (5%) were described as significant and reported.

## Results

### Characteristics of subjects

All of the samples were included in this study. The characteristics of subjects are shown in Table 1. There was significant variation found in maternal age (*p* < 0.05), systolic blood pressure (*p* < 0.05), diastolic blood pressure (*p* < 0.05), gestational age (*p* < 0.05), mode of delivery (*p* < 0.05), BMI (*p* < 0.05), and birth weight (*p* < 0.05). Patients with preeclampsia had highest maternal age 32.37 ± 6.41 years old, and highest BMI 26.80 ± 5.76. In contrast, preterm birth had the smallest BMI 21.78 ± 4.43 and lightest birth weight with 1937.5 g (1360.0–2370.0).

**Table 1:** Characteristics of subjects (n = 104).

Variables	Control (n = 44)	n (%)	Preeclampsia (n = 30)	n (%)	Preterm birth (n = 30)	n (%)
**Maternal age (years)**	29.77 ± 5.26		32.37 ± 6.41		28.27 ± 7.49**	
**Systolic BP (mmHg)**	120 (110–129.75)		180 (170–190.25)*		110 (110–120)*^,^**	
**Diastolic BP (mmHg)**	80 (70–80)		110 (100–130)*		70 (70–80)**	
**Gestational age at delivery**	39 (38–40)		36 (33–37.25)		33 (30–34)*^,^**	
Preterm (< 37 weeks)		0 (0)		16 (53.3)		30 (100)
Aterm (≥ 37 weeks)		44 (100)		14 (46.7)		0 (0)
**Mode of delivery**						
Vaginal delivery		20 (45.5)		1 (3.3)*		24 (80)*^,^**
C-section		25 (54.5)		29 (96.7)		6 (20)
**Body mass index (BMI)**	25.10 ± 4.52		26.80 ± 5.76		21.78 ± 4.43*^,^**	
Underweight (< 18.5)		4 (9.1)		1 (3.3)		6 (20)
Normal (18.522.9)		11(25)		8 (26.7)		13 (43.3)
Overweight (23–24.9)		6 (13.6)		4 (13.3)		6 (20)
Obese (≥ 25)		23 (52.3)		17 (56.7)		5 (16.7)
**Birth weight (g)**	3047.5 (2827.5–3437.5)		2377.5 (1762.5–3437.5)*		1937.5 (1360.0–2370.0)**	
Low (< 2500)		0 (0)		17 (56.7)		27 (90)
Normal-high (≥ 2500)		44 (100)		12 (43.3)		3 (10)

Data presented in Mean ± SD or Median (IQR).

*Significances to control (*p* < 0.05).

**Significances to preeclampsia (*p* < 0.05).

### LCPUFA levels in normal pregnancy, preeclampsia and preterm birth

Details of serum PUFA percentage and concentrations in control, preeclampsia and preterm birth are shown in Table 2. Preeclampsia was found to have the lowest percentage of Omega-3 derivates (ALA, EPA, and DHA). Regarding serum concentrations, it showed the highest number of total serum PUFA concentration (median 5896 (4862.5–7556.0) µmol/L) and lower amount of total omega-3 (median 345.00 (232.50–420.75) µmol/L compared to control. It also had the least amount of ALA (median 39.00 (25.75–64.00) µmol/L) and EPA (median 9 (6–13.5) µmol/L) compared to control and preterm birth. DHA percentage was significantly low among other groups (*p* < 0.05). In addition, preeclampsia group had the highest level of total omega-6 (median 5605 (4587.0–6976.25) µmol/L), LA (median 4725.5 (3882.0–5678.75) µmol/L), and AA (mean 607.5 ± 194.82 µmol/L). Also, having the highest ratio for omega-6/omega-3 (mean 17.40 ± 4.32) and AA/EPA (mean 66.5 ± 30.15) compared to other groups.

**Table 2:** Serum percentage and concentrations of LCPUFA (n = 104).

	Control (n = 44)	Preeclampsia (n = 30)	Preterm Birth (n = 30)
**PUFA**			
Concentration (µmol/L)	5088.5 (4622.75–6115.75)	5896 (4862.5–7556.0)*	4405.0 (3748.0–5308.5)*^,^**
**Total omega 3**			
Concentration (µmol/L)	391.50 (226.00–468.75)	345.00 (232.50–420.75)	331.5 (253.5–410.50)*
**ALA**			
Percentage (%)	0.42 (0.35–0.48)	0.22 (0.20–0.30)*	0.33 (0.28–0.40)*^,^**
Concentration (µmol/L)	59.5 (42.25–89.50)	39 (25.75–64.00)*	47 (29.75–55.50)*
**EPA**			
Percentage (%)	0.17 (0.10–0.23)	0.05 (0.04–0.08)*	0.15 (0.11–0.22)**
Concentration (µmol/L)	45.5 (18.00–31.75)	9 (6–13.5)*	18.5 (13–18.5)**
**DHA**			
Percentage (%)	2.12 ± 0.75	1.71 ± 0.45*	2.16 ± 0.73**
Concentration (µmol/L)	293 (244.50–374.75)	282 (204.75–338.75)	262 (198.75–339.25)
**Total omega 6**			
Concentration (µmol/L)	4624 (4221.5–5608.75)	5605.0 (4587.0–6976.25)*	4089 (3488.0–4836.75)*^,^**
**LA**			
Percentage (%)	27.15 ± 2.14	28.21 ± 1.97	26.08 ± 3.35**
Concentration (µmol/L)	3897 (3571.0–4636.5)	4725.5 (3882.0–5678.75)*	3382 (2868.5–4115.75)*^,^**
**AA**			
Percentage (%)	3.41 (3.07–4.45)	3.48 (3.06–3.93)	3.7 (2.90–4.43)
Concentration (µmol/L)	552.30 ± 129.90	607.5 ± 194.82	483.43 ± 141.72 *^,^**
**Omega 3 index**	3 (2–3)	2 (2–2)*	2.5 (2–3)**
**Omega 6/omega 3**	12.70 ± 3.39	17.40 ± 4.32*	12.53 ± 3.14*
**AA/EPA**	21.0 (17.25–29.00)	61.0 (42.50–89.75)*	26.5 (17.5–30.75)**

Data presented in Mean ± SD or Median (IQR).

*Significances to control (*p* < 0.05).

**Significances to preeclampsia (*p* < 0.05).

In contrast, preterm birth had the lowest total PUFA status (median 4405.0 (3748.0–5308.5) µmol/L) compared to other groups. It also had a low level of all of omega-3 and omega-6 parameters, particularly in total omega-3 (median 331.5 (253.5–410.50) µmol/L), ALA (median 47 (29.75–55.50) µmol/L), total omega-6 (median 4089 (3488.0–4836.75) µmol/L), LA (median 3382 (2868.5–4115.75) µmol/L), and AA (mean 483.43 ± 141.72 µmol/L, as it significantly low (*p* < 0.05) compared to control group.

### ROC curve analysis

**Figure 1:**
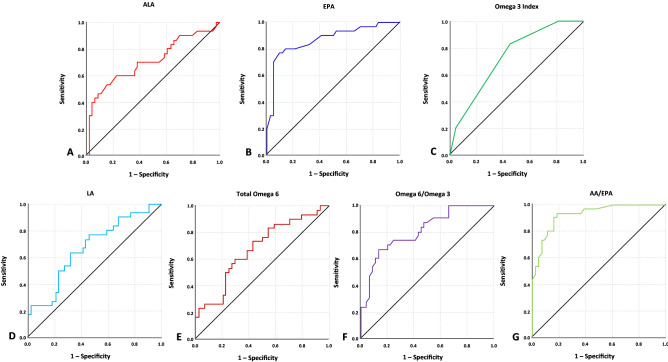
ROC curves of seven LCPUFA parameters which are considered to be acceptable predictors in showing the risk of severe preeclampsia by having AUC value > 0.650.

**Figure 2:**
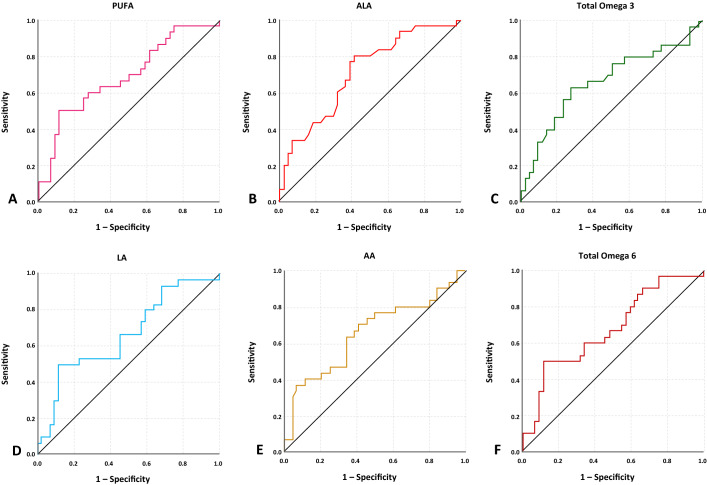
ROC curves of six LCPUFA parameters which are considered to be acceptable predictors in showing the risk of preterm birth by having AUC value > 0.650.

The AUC of ROC curves ranged 0.65–0.93 was taken into account, and all LCPUFA parameters had AUC < 0.65 were not included in further analysis as they are considered poor indicators for severe preeclampsia (Fig. 1 and Table 3) and preterm birth (Fig. 2 and Table 4). In predicting the risk of preeclampsia, EPA and AA had the highest AUC with 0.872 and 0.927. The best cut off points used with the sensitivity ranged 60.0–83.3% and specificity ranged 54.5–86.4%. The highest overall performance showed in ≤ 16 µmol/L EPA in having sensitivity 80% and specificity 86.4% in predicting the risk of preeclampsia, followed by AA/EPA with ratio ≥ 35, which had sensitivity 83.3% and specificity 84.1%. In regard of preterm birth, the lowest AUC value which included for further analysis was 0.654 for AA and the highest was 0.709 for ALA. For sensitivity and specificity analysis in preterm birth risk, the best cut off points used with the sensitivity ranged 53.3–66.7% and specificity ranged 63.6–77.3%.

**Table 3:** Predictive value of seven LCPUFA parameters in showing the risk of severe preeclampsia.

Variables	AUC	95%CI	Optimal cut-off value	Sensitivity (%)	Specificity (%)
ALA	0.706	0.578–0.833	53	63.3	63.6
EPA	0.872	0.784–0.959	16	80.0	86.4
Omega-3 index	0.731	0.618–0.844	2.5	83.3	54.5
Total omega-6	0.679	0.554–0.803	5194	60.0	70.5
LA	0.670	0.544–0.795	4370	60.0	68.2
Omega-6/omega 3	0.806	0.705–0.906	14.5	73.3	65.9
AA/EPA	0.927	0.869–0.984	35	83.3	84.1

**Table 4:** Predictive value of six LCPUFA parameters in showing the risk of preterm birth.

Variables	AUC	95%CI	Optimal cut-off value	Sensitivity (%)	Specificity (%)
PUFA	0.687	0.562–0.813	4687	60.0	72.7
Total omega-3	0.666	0.534–0.798	345	63.3	72.7
ALA	0.709	0.590–0.828	53	66.7	63.6
Total omega-6	0.674	0.548–0.801	4354	60.0	65.9
LA	0.666	0.538–0.794	3533	53.3	77.3
AA	0.654	0.521–0.787	500	63.3	65.9

### Bivariate and multivariate analysis

Furthermore, bivariate and multivariate analysis were performed using the cut off points for predicting preeclampsia and preterm birth risk. Serum concentrations of EPA (*p* < 0.001), ALA (*p* = 0.033), Omega-3 index (*p* = 0.002), total omega-6 (*p* = 0.018), LA (*p* = 0.030), Omega-6/omega-3 (*p* = 0.004) and AA/EPA (*p* < 0.001) were found to be significant in between preeclampsia and control. The overall high omega-6 and low omega-3 content in preeclampsia were shown as the risk increase with high Omega-6/Omega-3 ratio (OR 10.02, 95%CI 1.17–56.66), along with low omega-3 index (OR 12.19, 95%CI 1.65–89.64) (Table 5). In addition,

**Table 5:** LCPUFA parameters showing the risk of preeclampsia.

Variables	Preeclampsia	Control			
	n (%)	n (%)	P bivariat	Unadjusted OR (95%CI)	Adjusted OR (95%CI)*
**ALA (µmol/L)**					
High risk (≤ 53)	20 (66.7)	17 (38.6)	0.033	3.17 (1.20–8.39)	5.44 (1.16–25.42)
Low risk (> 53)	10 (33.3)	27 (61.4)		1.0	1.0
**EPA (µmol/L)**					
High risk (≤ 16)	24 (80)	9 (20.5)	< 0.001	15.55 (4.89–49.43)	30.84 (4.08–232.61)
Low risk (> 16)	6 (20)	35 (79.5)		1.0	1.0
**Omega-3 index**					
High risk (≤ 2.5)	25 (83.3)	20 (45.5)	0.002	6.00 (1.94–18.55)	12.19 (1.65–89.64)
Low risk (> 2.5)	5 (16.7)	24 (54.5)		1.0	1.0
**Total Omega-6 (µmol/L)**					
High risk (≥ 5194)	18 (60)	13 (29.5)	0.018	3.57 (1.34–9.48)	1.99 (0.45–8.80)
Low risk (< 5194)	12 (40)	31 (70.5)		1.0	1.0
**LA (µmol/L)**					
High risk (≥ 4370)	18 (60)	14 (31.8)	0.030	3.21 (1.22–8.45)	1.68 (0.38–7.51)
Low risk (< 4370)	12 (40)	30 (68.2)		1.0	1.0
**Omega-6/Omega 3**					
High risk (≥ 14.5)	22 (73.3)	16 (36.4)	0.004	4.81 (1.74–13.29)	10.02 (1.17–56.66)
Low risk (< 14.5)	8 (26.7)	28 (63.6)		1.0	1.0
**AA/EPA**					
High risk (≥ 35)	26 (86.7)	7 (15.9)	< 0.001	34.35 (9.11–129.50)	24.78 (4.25–144.27)
Low risk (< 35)	4 (13.3)	37 (84.1)		1.0	1.0

* Adjusted for covariates: Maternal age, Gestational age, and Maternal pre-pregnancy BMI.

1.0: Reference Value.

For the analysis showing preterm birth risk (Table 6), an overall low concentration of PUFA (*p* = 0.010) found to be significant compared to control. Accompanied by the increase risk in low omega-3 (OR 3.10, 95%CI 1.11–8.67) and low omega-6 (OR 3.00, 95%CI 1.07–8.40).

**Table 6:** LCPUFA parameters showing the risk of preterm birth.

Variables	Preterm birth	Control			
	n (%)	n (%)	P bivariate	Unadjusted OR (95%CI)	Adjusted OR (95%CI)*
**PUFA (µmol/L)**					
High risk (≤ 4687)	18 (60)	12 (27.3)	0.010	4.0 (1.49–10.73)	4.09 (1.42–11.82)
Low risk (> 4687)	12 (40)	32 (72.7)		1.0	1.0
**Total omega-3 (µmol/L)**					
High risk (≤ 345)	19 (63.3)	15 (34.1)	0.025	3.33 (1.26–8.80)	3.10 (1.11–8.67)
Low risk (> 345)	11 (36.7)	29 (65.9)		1.0	1.0
**ALA (µmol/L)**					
High risk (≤ 53)	20 (66.7)	17 (38.6)	0.033	3.17 (1.20–8.39)	4.68 (1.52–14.38)
Low risk (> 53)	10 (33.3)	27 (61.4)		1.0	1.0
**Total Omega-6 (µmol/L)**					
High risk (≤ 4354)	18 (60)	15 (34.1)	0.050	2.90 (1.11–7.57)	3.00 (1.07–8.40)
Low risk (> 4354)	12 (40)	29 (65.9)		1.0	1.0
LA (µmol/L)					
High risk (≤ 3533)	16 (53.3)	10 (22.7)	0.014	3.88 (1.42–10.62)	4.42 (1.46–13.35)
Low risk (> 3533)	14 (46.7)	34 (77.3)		1.0	1.0
**AA (µmol/L)**					
High risk (≤ 500)	19 (63.3)	15 (34.1)	0.025	3.33 (1.26–8.80)	2.79 (1.00–7.77)
Low risk (> 500)	11 (36.7)	29 (65.9)		1.0	1.0

*Adjusted for covariates: Maternal age and Maternal pre-pregnancy BMI.

1.0: Reference Value.

In comparing 2 variable outcomes, serum concentration of ALA ≤ 53 µmol/L have shown to increase the risk of preeclampsia by OR 5.44, 95%CI 1.16–25.42, with sensitivity 63% and specificity 63.6%, and the risk of preterm birth by OR 4.68, 95%CI 1.52–14.38, with sensitivity 66% and specificity 63.6%.

## Discussion

This research was dominated by overweight to obese participants. This findings was in conjunction with previous study in Indonesia, where 49.1% of 234 first trimester pregnant women were also found to have BMI ≥ 23^12^. We also found that preterm birth group had the lightest mean of BMI. This data was supported by reviews and meta-analysis, which suggested that low maternal BMI is associated with preterm birth^23^.

In comparing LCPUFA concentrations of those 3 groups, preterm birth group had the lowest total LCPUFA, total omega-3, and omega-6. Even though studies have shown that there is an increasing trend of LCPUFA level in serum, erythrocyte and plasma throughout pregnancy^24–26^, studies have also suggested that low total omega-3 had increased the risk of preterm birth < 34 weeks, which can be reduced through LCPUFA supplementation^27,28^. In addition, gestational age seems not affecting total LCPUFA content in preeclampsia, as being the highest level among others. This was thought to influenced by numerous amounts of omega-6 serum concentration.

Preeclampsia and preterm birth group found to have lower amount of omega-3 PUFA than control group, with preterm and control group showed a significant difference (*p* < 0.05). This is consistent with other findings, where low number of both omega-3 and omega-6 PUFA in preeclampsia and preterm were found^15,29^. Omega-3 fatty acids, including EPA and DHA, have a proangiogenic effect in extra villous trophoblast cell lines, hence increase the development of capillary sprouts^30^. Studies have shown that supplementation of omega-3 fatty acids during pregnancy may improve birth weight as well as decrease preterm birth rate^27,31^. Therefore, a good counselling to encourage pregnant women in improving omega-3 dietary intake and supplementation are required.

ALA and EPA are essential omega-3 fatty acids which have anti-inflammatory properties and vascular benefit. In our study, we found that both preeclampsia and preterm birth have lower ALA and EPA serum concentration compared to control. These were shown by EPA concentrations ≤ 16 µmol/L was significantly associated with preeclampsia (*p* < 0.001), followed by ALA maternal serum concentration ≤ 53 µmol/L have shown to increase the risk of both pregnancy complications (preeclampsia by OR 5.44, 95%CI 1.16–25.42 and preterm birth by OR 4.68, 95%CI 1.52–14.38). These findings were consistent with other studies where low ALA and EPA were associated with higher preeclampsia risk^29,32^. Moreover, a study by Stewart et al. showed lower ALA was found in patients with earlier gestational age as ALA is expected to increase along pregnancy and become the precursor for omega-3 metabolites, which are EPA and DHA^26^. Even though the conversion process is not efficient in human and depend on epigenetic, genetic process, as well as sex^33,34^, another study suggest that the level of maternal ALA is associated with DHA concentration in blood cord^35^. A nutrition study about dairy product with ALA, LA, and DHA found to reduce the rate of early preterm birth and improve the length of gestation^33^. Thus, pregnant women are advised take sufficient amount of ALA and EPA on early terms, using supplementations or omega 3 rich food such as soybean oil, canola oil, green vegetables, and seafood^36,37^.

Furthermore, DHA percentage was found to be significantly low for preeclampsia compared to control (*p* > 0.05) and preterm birth (*p* < 0.05). A study have shown that DHA may decrease oxidative stress in deep placentation disorders, including preeclampsia and preterm birth^30^. Another study also showed DHA supplementation during pregnancy significantly reduce the risk of preeclampsia and severe preeclampsia^38^. In addition, preterm subjects showed the least of DHA concentrations result among groups and EPA result was lower compared to control group. Studies have shown that DHA promotes longer gestation period by acting as prostaglandin inhibition for ripening cervix and slowing uterine contraction in last week pregnancy^39^. Trial by Carlson et al. showed supplementation of 600 mg DHA daily given at early pregnancy resulted in significant longer pregnancy (*p* = 0.041) and higher birthweight (*p* = 0.004)^40^. Other study supported this result by showing a decreased preterm birth rates in patients taking Omega-3 PUFA levels up to ~ 600 mg daily^41^.

Omega-3 index, which is a combination of EPA and DHA level, is suspected to represent functional PUFA status. Our data also showed that preterm birth had 2.5% omega-3 serum index. This result was close to findings by Olsen et al., which proposed that the risk of preterm labor increase if the value of plasma PUFA below 2.0%, with two measurements of samples in the early pregnancy^15^. Our results also showed that cut off point 2.5% was suitable for predicting the risk of preeclampsia with sensitivity 83% and specificity 54.5%, following the increased risk by OR 12.19, 95%CI 1.65–89.64. Therefore, high omega-3 diet as well as supplementation such as DHA, should be considered for pregnant women.

In addition, we found significantly higher level of total omega-6 PUFA (*p* < 0.05), LA (*p* < 0.05), and AA (*p* < 0.05) in preeclampsia compared to others. These findings were accompanied with lower total omega-3 level along with its derivates (ALA, EPA and DHA) in preeclampsia compared to control group. The result also showed significantly higher ratio (*p* < 0.05) of omega-6/omega-3 in preeclampsia group compared to control group. This study showed an altered condition of fatty acid level that occur in preeclampsia patient as shown in previous studies before^42,43^.

Our data did not show causality LCPUFA status on preeclampsia, however our result on high total omega-6 level and concordance to high ratio of omega-6/omega-3 may play role in inflammatory reaction in preeclampsia pathogenesis, thus further study is required^6,44^. Higher omega-6 with low omega-3 has been previously associated with promotion of platelet aggregation, vasoconstriction and proinflammatory along with prothrombotic state which contribute to preeclampsia pathophysiology^45,46^. This is in conjunction with our results where both of the pathological conditions had significantly higher value of omega-6/omega-3 ratio compared to control (*p* < 0.05). Meanwhile, preterm birth had the lowest mean of omega-6/omega-3 ratio. This might be resulted by relatively low total of omega-3 with relatively normal omega-6 level and lower in its derivates such as AA in preterm group.

Our study found that there was a significantly high level of AA/EPA ratio in preeclampsia compared to other groups. Previous studies have suggested that AA/EPA have positive correlation with inflammatory rate of eicosanoids^47,48^, particularly due to AA eicosanoid metabolites^30,49^. On the other hand, AA is also found to generate lipoxins, particularly lipoxin (LXA4), which acts as an anti-inflammatory properties that related to obstetrical complications such as preterm and preeclampsia^50,51^. Here we showed that the value of ≥ 35 AA/EPA has increased the risk for preeclampsia with sensitivity 83.3% and 84.1%, with significant differences compared to control (*p* < 0.001) and high adjusted odds ratio (OR 24.78). However, the wide 95%CI (4.25–144.27) found in our study suggesting that this optimal value of AA/EPA should be further elaborated, followed by other metabolites analysis.

Certain limitations need to be considered to interpret our study. We did not asses daily intake of PUFA, as well as other biomarkers which responsible in pathological mechanism related to obstetrical outcomes. We also unable to analyse non-fasting blood samples as preeclampsia and preterm birth maternal condition which required highly nutrient intake. Suggesting that mothers who are in labor, especially with vaginal delivery method, should be energized enough to undergo the process. In addition, triglyceride elimination before trans-esterification was unable to performed. Thus, the calculated fatty acids were both free fatty acids and fatty acids which might still bind to triglycerides and phospholipid. Nevertheless, this initial research throughout this topic could represent an overview of maternal LCPUFA status in pregnant women with preeclampsia and preterm birth, as well as a beginning for further research. Detail evaluation of nutritional intake, LCPUFA supplementation, other metabolites as well as sample processing with wider range of population are required in order to gain better understanding regarding LCPUFA effect on obstetrical outcome.

## Conclusion

Our study proposed that both severe preeclampsia and preterm birth have an imbalance in fatty acid metabolism. LCPUFA plays a great role in those pathological conditions, thus dietary balanced intake of omega-3 and omega-6 enriched functional food or supplementation in pregnant women should be highly considered.

## Data Availability

The datasets used and/or analysed during the current study are available from the corresponding author on reasonable request.

## Abbreviations

LCPUFA: Long-chain polyunsaturated fatty acid
PUFA: Polyunsaturated fatty acid
ALA: Alpha-linolenic acid
EPA: Eicosapentaenoic acid
DHA: Docosahexaenoic acid
LA: Linoleic acid
AA: Arachidonic acid
GC-MS: Gas chromatography/mass spectrometry
LXA4: Lipoxin A4
